# Dietary and lifestyle interventions for the management of hereditary ataxias

**DOI:** 10.3389/fnut.2025.1548821

**Published:** 2025-04-24

**Authors:** Wenyao Yang, Bruce Thompson, Faith A. A. Kwa

**Affiliations:** ^1^School of Health Sciences, Swinburne University of Technology, Hawthorn, VIC, Australia; ^2^Melbourne School of Health Sciences, The University of Melbourne, Parkville, VIC, Australia

**Keywords:** Ataxia telangiectasia, dietary changes, Friedreich Ataxia, hereditary ataxia, lifestyle interventions, nutraceuticals, spinocerebellar ataxia

## Abstract

Hereditary ataxia (HA) is a diverse group of rare inherited neurological disorders characterised by cerebellar impairment and the progressive degeneration of spinocerebellar tracts and the spinal cord. These conditions manifest predominantly as unsteady gait, speech difficulties, dysphagia and motor skill impairment. The complex genetic causes and varied disease mechanisms underlying HA contribute to the multi-systemic symptoms which pose challenges in developing targeted effective treatments. Currently, available options for HA primarily focus on symptomatic management, highlighting a critical need for complementary therapeutic strategies, such as dietary and lifestyle interventions. This review explains recent findings on dietary and nutraceutical interventions, as well as lifestyle modifications such as exercise and rehabilitation programs for HA. It outlines common types of HA, including Friedreich ataxia, spinocerebellar ataxias, ataxia with vitamin E deficiency, ataxia-telangiectasia, and studies on a mixed cohort of patients with HA. The current management options, therapeutic implications of findings from pre-clinical and clinical data and future directions to advance the treatment of HA will also be discussed. The integration of nutraceuticals and rehabilitation programs with current methods of symptomatic management is encouraged for the holistic treatment of HA. These interventions will complement the use of various technological aids with the support of a multidisciplinary health and medical team to improve monitoring of the health status and disease progression of affected individuals; thus facilitating early treatment and an optimised clinical outcome.

## Introduction

Hereditary ataxia (HA) is a group of genetic neurodegenerative disorders characterised by the progressive loss of coordination and balance due to the degeneration of the central nervous system (1). HA can be categorised into various subgroups, with autosomal recessive and autosomal dominant inheritance patterns being the most studied (1). More than 40 types of autosomal dominant HA, often referred to as spinocerebellar ataxias (SCAs), typically exhibit onset during adulthood (2). In contrast, autosomal recessive ataxias generally present in childhood, with common subtypes including Friedreich ataxia (FRDA), ataxia-telangiectasia (AT), and ataxia with vitamin E deficiency (AVED) (1). These ataxias share common symptoms such as unsteady gait, difficulties with speech and swallowing, and a wide range of other neurological symptoms, ultimately impairing the independence and quality of life of affected individuals (3).

To date, amongst all HAs, FRDA is the only subtype with an FDA-approved treatment (i.e., omaveloxolone) (4). Despite not being clinically approved, high-dose vitamin E supplementation has been widely recommended for treating AVED, making it a “dietary treatable condition” (5). Nevertheless, for the remaining types of HA, symptomatic management and supportive care remain the primary treatment options (3). This underscores the pressing need for complementary therapeutic strategies including dietary and lifestyle interventions to bring hope of an improved quality of life for those affected by HA.

This review discusses the pre-clinical and clinical findings related to dietary and nutraceutical interventions, as well as lifestyle modifications, including exercise and rehabilitation programs, for the aforementioned types of HA. In addition, this review highlights the potential benefits and challenges of these interventions in managing HA.

## Search strategy and study selection criteria

Due to a small number of articles published on this research topic and in the effort to critique the findings of relatively recent studies, a literature search was conducted to identify peer-reviewed original research articles published within the last 10 years. These articles focused on dietary interventions, nutraceuticals, and lifestyle modifications for the management of hereditary ataxias. Keywords used included “Hereditary ataxia,” “Spinocerebellar Ataxia,” “Friedrich Ataxia,” “Ataxia-telangiectasia,” and “Ataxia with vitamin E deficiency” “physical rehabilitation,” “physical activity,” “dietary interventions,” “nutraceuticals,” “exercise,” and “training.” The search was performed using PubMed, and Web of Science. Full-text articles that met the above criteria were then assessed for their relevance to the objectives of this review.”

## Features of hereditary ataxias

Despite sharing the above common clinical manifestations, HA possesses distinct pathogenic pathways and unique disease features. Due to genetic heterogeneity, HA can be a multigenic condition (2, 3). These mutations can impact various aspects of neuronal function, resulting in a wide range of symptoms and differing rates of disease progression (6). While primarily neurological, many HAs also present with non-neurological symptoms that can involve multiple organs, including retinopathy, cardiomyopathy, myopathy, and disorders associated with the endocrine and immune systems (7, 8). For certain types of HA associated with polymorphic tandem repeats, clinical features can vary significantly based on the size of the repeat expansion (9, 10). Depending on their sequence and location, repeat expansions may lead to either a loss- or gain- of-function, affecting gene expression and protein functionality (9). Additionally, specific gene mutations can also result in cellular changes with functional consequences within the nervous systems. These will be discussed in the following subsections.

## Autosomal recessive hereditary ataxias

FRDA is caused by the mutation of guanine-adenine-adenine (11) repeat expansion in the *frataxin* (*FXN*) gene, which encodes a protein lead to a loss of function that is essential for mitochondrial iron homeostasis and the assembly and transfer of iron–sulfur clusters (11, 12). The mutation results in FXN deficiency, leading to mitochondrial dysfunction and symptoms such as gait ataxia, sensory loss, scoliosis and potential cardiac complications (12). Although symptoms typically reveal around puberty, the age of onset and the disease severity are inversely correlated with the size of the GAA expansion on the smaller of the FXN alleles (13).

A-T is caused by mutations in the *Ataxia Telangiectasia Mutated* (*ATM*) gene, which is crucial for the repair of DNA double-strand breaks and maintenance of genomic stability (14). Due to the defects in DNA repair, individuals with A-T have an increased risk of cancer and immunodeficiency (15). It is characterised by progressive cerebellar ataxia, oculomotor apraxia, or uncoordinated eye movements between ages 1 and 4 (1, 3, 15).

AVED is caused by mutations in the *TTPA* gene, which encodes the alpha-tocopherol transfer protein critical for vitamin E transport (1, 5). This condition is characterised by ataxia and peripheral neuropathy beginning in early childhood due to impaired vitamin E distribution in cells and tissues (1). Symptoms including dysarthria and poor balance in dim lighting result from the early loss of proprioception. As the condition progresses, many individuals become wheelchair-bound due to ataxia or leg weakness between the ages of 11 and 50 (1). While AVED shares phenotypic similarities with FRDA, it has distinct features such as head titubation, and dystonia with reduced frequency of cardiomyopathy (1, 5).

## Autosomal dominant hereditary ataxias

The most extensively studied SCAs, characterised by a variable cytosine-adenine-guanine (CAG) expansion within the coding region of their respective genes directly results in the formation of an extended polyQ tract in the encoded proteins, leading to conformational changes that confer a toxic gain of function (6, 16). Uninterrupted (pure CAG) repeat structures increase the risk of expansions whereas CAT interruptions may stabilise these repeats during transmission and enhance meiotic stability (2). The larger the expansion size, the earlier the age of onset and the more severe the disease phenotype are observed in SCA (16). SCA1 is caused by a CAG repeat mutation in the *Ataxin-1 (ATXN1)* gene, resulting in toxic polyQ expansion that leads to a predominant loss of large populations of neurons in the cerebellum, brainstem, spinal cord, and cerebral cortex, with affected individuals experiencing pyramidal symptoms and muscle atrophy, and respiratory failure being the main cause of death (7, 16, 17). Other SCAs, such as SCA2, SCA3, SCA6, SCA7, and SCA17, are linked to polyQ expansions in distinct proteins, including Ataxin-2 (ATXN2), Ataxin-3 (ATXN3), calcium voltage-gated channel subunit alpha 1 (CACNA1A), Ataxin-7 (ATXN7) and the TATA binding protein (3). Saccadic slowing/saccadic dysmetria, nystagmus, and symptoms related to peripheral neuropathy are frequently seen in affected individuals with SCA2 (16). Notably, repeat sizes greater than 45 are almost always associated with a more aggressive disease course and disease onset before the age of 20 years for SCA2 (7, 18). SCA3, or Machado-Joseph disease, is the most prevalent SCAs worldwide. The protein aggregation leads to the formation of intranuclear inclusions, resulting in neuronal loss and specific symptoms such as pyramidal signs, parkinsonism, dystonia (16, 19, 20). SCA6 is characterised by progressive ataxia and postural instability due to mutations affecting calcium channels in the cerebellum (16). A recent study highlighted that heterozygous loss-of-function variants in the *CACNA1A* gene not only result in classical ataxia but are also associated with a range of other phenotypes, including epilepsy and intellectual disability (21). SCA7 is associated with vision problems (i.e., slow saccadic eye movement, ophthalmoplegia) due to retinal degeneration in addition to ataxia (16). Mental deterioration, occasional chorea, dystonia, myoclonus, epilepsy are often seen in SCA17 (1, 16). Unlike the above-mentioned common SCA subtypes, SCA38 is caused by missense mutation in the Elongase of Very Long-chain fatty acids 5 (*ELOVL5*) gene, which is necessary for synthesising omega-3 and omega-6 polyunsaturated fatty acids (PUFAs). It is characterised by a progressive and debilitating condition marked by ataxia, hyposmia, peripheral neuropathy, and cerebellar atrophy. Individuals with SCA38 may lose their ability to walk or may need to rely on assisted feeding within three decades of diagnosis (22).

## Current disease management and novel therapeutic options

The pathogenesis underlying HA is complex, addressing the need of a multifaceted therapeutic strategy that can target the genetic cause, regulation of mitochondrial function and metabolism, and are compatible with methods of managing symptoms.

Current therapeutic options for the HA are significantly limited by both availability and the efficacy of available drugs. Many therapies are not widely accessible or approved in all countries or for all age groups. For instance, the first FDA-approved drug for FRDA, Omaveloxolone (SKYCLARYS™), is restricted to individuals under 16 years of age in the countries of US and EU (4). Regarding its efficacy, as a nuclear factor erythroid 2-related factor 2 (Nrf2) activator, it can help improve mitochondrial function, restore redox balance and reduce inflammation; however, it was not designed to target mutation in the *FXN* gene and improve FXN protein abundance (4).

While potential treatments are currently under investigation, the efficacy of these therapies can vary significantly among individuals due to genetic differences, disease progression, and other comorbidities (3, 23). Various therapeutic approaches, including gene therapy, protein replacement therapy, are being developed (24). However, advancement of these approaches in clinical trials is often hindered by a lack of relevant pre-clinical models (24). For example, while antisense oligonucleotides (ASOs) have demonstrated efficacy in mouse models of SCAs (25, 26). Those models are inadequate to fully recapitulate the full spectrum of disease severity seen in humans, such as neurodegeneration and cardiomyopathy seen in late-stage disease (24). Moreover, despite promising pre-clinical findings associated with molecular therapies, the translation of such data in the clinical setting is questionable due to a small clinical trial community for HA, short follow-up periods, varied outcome measurements and lack of reliable predictors of clinical outcomes. Consequently, these factors can lead to non-reproducible results which challenge the efficacy of new treatments (13, 27).

There is currently no clinically approved pharmacologic therapy for most types of HA. Supportive care and symptomatic treatments, such as physical therapy and medications for muscle spasticity, are the main interventions. Nevertheless, managing ataxic disorders is often difficult due to significant heterogeneity and the presence of symptoms affecting multiple organs. To effectively manage these complex conditions, affected individuals often require a multidisciplinary team comprising neurologists, physiotherapists, and genetic counselors to address the multifaceted symptoms and associated comorbidities (27). For instance, speech therapy is recommended to manage dysarthria and swallowing difficulties, while psychological support plays a crucial role in addressing emotional and cognitive challenges faced by affected individuals. Regular assessments are necessary to monitor disease progression and adjust treatment plans as needed (28).

To address the above concerns, there is an urgent need for the development of safe, accessible and complementary therapeutic strategies such as dietary and lifestyle interventions.

## Dietary interventions and nutraceuticals for individuals with hereditary ataxias

Natural products (i.e., nutraceuticals) have had a long-standing reputation as valuable candidates in drug discovery, yielding exciting findings in alleviating symptoms and preventing disease progression in various conditions (29). Extensive evidence from different studies highlights the benefits of nutraceuticals, such as vitamin supplementation and phytochemicals, in treating HA (Table 1) (22, 30–32).

**Table 1:** Dietary interventions and nutraceuticals for the treatment of hereditary ataxias.

Type of hereditary ataxia	Type of study (clinical trial, in vivo, in vitro studies)	Treatment regimen	Key findings	Reference
Autosomal Recessive Hereditary Ataxias				
Friedreich Ataxia	Clinical trial: 34 participants	100 mg of thiamine was administered intramuscularly on a twice weekly basis for 80–930 days.	Improved SARA score, deep tendon reflexes, swallowing, interventricular septum thickness compared to the baseline. No adverse events. FSS scores, *FXN* blood expression did not change.	Costantini et al. (33)
Friedrich Ataxia	Clinical trial: 10 participants	Phase 1: Oral nicotinamide single doses of 0, 2, 4, 6, 7 and 8 g for 5 visits. Phase 2: Daily doses with dose escalation 3.5-6 g for 5 days. Phase 3: Daily doses at maximum tolerated dose <6 g for 8 weeks.	Increased the frataxin protein levels via epigenetic modification. No significant improvements observed in clinical measures (SARA, SCAFI, Speech Intelligibility Test, ADL). Well-tolerated, with mild nausea occurring at high doses.	Libri et al. (35)
Friedreich Ataxia	Case report: 1 individual	4.4 g of nicotinamide was taken daily for 10 days.	Severe adverse reactions such as nauseous, tired, vomiting and loss of consciousness. Caused life-threatening shock, lactic acidosis and hepatic failure.	Garin and Arnold (36)
Friedrich Ataxia	*In vivo*: Knockdown of frataxin (shFxn) transgenic mice (disease) C57BL/6J WT mice (control)	3.45 mg/ml of nicotinamide mononucleotide (NMN) and 3 mg/ml of nicotinamide riboside (NR) were administered in drinking water for up to 26 weeks.	Improved the survival of mice, particularly in males, modestly improved cardiac hypertrophy, and limited the increases in ejection fraction along with increased glutathione levels in the heart tissue. Partially restored metabolic imbalances but had no effect on transcriptional levels. Weight, motor coordination, insulin levels were not affected by the treatments.	Perry et al. (34)
Friedreich Ataxia	*In vitro*: DRG neurons and cardiomyocytes from Frataxin-deficient rats Lymphoblastoid cells from FRDA patients	20 nM of calcitriol was treated for DRG neurons and cardiomyocytes over 5 days and 100 nM of calcitriol was incubated for lymphoblastoid cells for the same duration.	Increased frataxin protein levels in all cell types. Restored ΔΨm and mitochondrial calcium exchanger levels, reduced calcium overload and improved mitochondrial morphology. Reduced neuronal apoptosis and neurite degeneration.	Britti et al. (39)
Friedreich Ataxia	Clinical trial: 15 participants	0.25mcg/day of calcitriol administered for 1 year.	Failed to improve neurological function (SARA, 9-HPT, 8-MWT, PATA) but increased FXN levels in platelets at 5.5–7.0 pg./μg dose range. 5 out of 20 participants discontinued due to mild hypercalcemia.	Alemany-Perna et al. (30)
Friedreich Ataxia	*In vitro*: CGNs, cardiomyocytes from YG8R mice (disease) CGNs, cardiomyocytes from Y47R mice (control)	200 μM of vitamin E was added for 30 min under pre-hypoxic/reperfusion conditions for both control and disease cells.	Restored calcium hemostasis in cardiomyocytes. Prevented cell death induced by hypoxia-reperfusion injury.	Abeti et al. (10)
Friedreich Ataxia	Clinical trial: 5 FRDA patients and 7 heathy individuals	5 m/kg/day of tocotrienol was supplied as an add-on therapy to idebenone (5 mg/kg body wight/day) for 12 months.	Increased GSH/GSSG ratio and various antioxidant gene markers, reduced levels of plasma protein carbonylation and inflammation markers. Improved cardiac CMR and lipid profile were found at 2 and 12-month post-treatment. Increased oxygen radical absorbance capacity only after 12-month post-treatment.	Bolotta et al. (41)
Friedreich Ataxia	*In vitro*: Visceral white adipose tissue from KIKO mice (disease) Visceral white adipose tissue from WT mice (control)	5 g/kg/day of sodium butyrate in diet or a normal diet was fed to 4-month-old mice for a duration of 16 weeks.	Reduced inflammation and improved glucose tolerance, adipocyte metabolism, and restored mitochondrial respiratory capacity and the abundance of butyrate-producing bacteria in the gut compared to the untreated disease group.	Turchi et al. (42)
Friedrich Ataxia	*In vivo*: FXN¯ mutant mice (disease) WT mice (control)	0.0185 mg/g or 0.185 mg/g of the epigallocatechin gallate (EGCG) was administrated until the age of 6 months.	Treated mice counteracted the delay in cerebellar neurogenesis (size and white matter volume of the spinal cord, increased β-tubulin expression). Partially restored neurite outgrowth (increased Contactin 1 expression). Low dose EGCG increased glial fibrillary acidic protein expression, high dose did the opposite.	Bizzoca et al. (43)
Friedrich Ataxia	*In vitro*: YG8R FRDA mouse fibroblasts KIKO FRDA mouse fibroblasts	Pre-treated with 50 nM sulforaphane (SFN) for 24 h or untreated for control, then exposed to 1 mM H_2_O_2_ for 2 h to induce oxidative stress.	Prevented cell death in both cell models. Reduced H_2_O_2_ induced lipid peroxidation in both cell models. Rescued the depolarization in YG8R cells but not seen in the KIKO cells.	Abeti et al. (45)
Friedrich Ataxia	*In vitro*: FRDA fibroblasts	10 μM SFN treatment for 24 h	Rescued ferroptosis markers (decreased lipid peroxidation and increase glutathione peroxidase 4).	La Rosa et al. (46)
Friedrich Ataxia	*In vitro*: Neural stem cells from KIKO mice (disease) Neural stem cells from WT mice (control)	5 μM SFN treatment for 2,6 or 24 h.	Increased Nrf2 and its target gene expression, leading to increased proliferation, clonogenicity, and neuronal complexity, reduced ROS production	La Rosa et al. (44)
Friedrich Ataxia	*In vitro*: shFxn neurons FRDA fibroblasts	5 μM SFN in neurons and 10 μM SFN in FRDA fibroblasts both treated for 24 h.	Increased Nrf2 expression and the expression of its downstream target in both cell models. Restored the redox balance in shFxn neurons by decreasing GSSG and increasing GSH with increased FXN levels and improved neurite outgrowth in shFxn neurons.	Petrillo et al. (47)
Friedrich Ataxia	*In vitro*: FRDA fibroblasts	10 μM SFN for 24 h.	Increased FXN gene expression. Increased NRF2 expression and Nrf2 target genes.	Petrillo et al. (48)
Ataxia-telangiectasia	*In vitro*: AT fibroblasts (disease) HT fibroblasts (control) ATM-deficient SH-SY5Y5 neuronal cells (disease) SH-SY5Y5 neuronal cells (control) *In vivo*: Atm−/− mice (disease) WT mice (control)	1 mM of nicotinamide riboside (NR) treatment or vehicle treatment for 24 h or 10 days for *in vitro*, 2 months for *in vivo*.	Increased NAD + levels, suppressed cellular senescence and neuroinflammation (IL6 and IL1β). Improved mitochondrial functions and reduced cytoplasmic dsDNA in neuronal cells. Prevented neurodegeneration and senescence, improved mitochondrial homeostasis and motor function.	Yang et al. (37)
Ataxia-telangiectasia	*In vivo*: atm-1 *C. elegans* (disease) N2 *C. elegans* (control) Atm−/−KD mice (disease) WT mice (control)	500 μM of nicotinamide riboside (NR) or vehicle for treating *C. elegans* across the lifespan; 12 mM of NR or nicotinamide mononucleotide (NMN) was administered in drinking water for 14 days for the mice. Control mice received only drinking water.	Reduced the severity of A-T neuropathology, restored neuromuscular function and metabolomics profiles, reduced memory loss and improved lifespan in both animal models.	Fang et al. (38)
Ataxia with vitamin E deficiency	*In vitro*: DRGNs from Ttpa−/− mice (disease) DRGNs from Ttpa+/+ mice (control)	Control mice were fed a normal α-tocopheryl acetate diet (35 mg); disease mice were fed with *α*-tocopheryl acetate deficient diet (<10 mg) or high α-tocopheryl acetate diet (600 mg) for 6 months.	Vitamin E deficient cells showed electrophysiological abnormalities, resulting in cell apoptosis. High vitamin E diet prevented the cellular and electrophysiological alterations and improved mechanosensation in disease model.	Finno et al. (49)
Ataxia with vitamin E deficiency	*In vivo*: Ttpa−/− mice (disease) Ttpa+/+ mice (control)	Control mice were fed a normal vitamin E diet (75 mg synthetic); disease mice were fed with vitamin E deficient diet (undetectable) or natural vitamin E diet (600 mg), synthetic vitamin E diet (816 mg), or high synthetic vitamin E diet (1,200 mg) for 4 weeks.	All natural and synthetic vitamin E treatment groups found to increase α-tocopherol levels in the brain, heart and lung tissues compared to the disease group. High-dose synthetic vitamin E may have negative impact on myelination.	Ranard et al. (50)
Ataxia with vitamin E deficiency	Clinical trial: 1 participant	1.8 g of oral ɑ-tocopherol acetate was given daily in three doses.	Kept the plasma levels constantly in a supernormal range of 20–40 mg/L. No clinically detectable progression of the neurodegenerative process, no “loading effect” developed.	Kohlschutter et al. (51)
Autosomal Dominant Hereditary Ataxias				
Spinocerebellar Ataxia type 3 or Machado–Joseph disease	*In vitro*: Ataxin-3 protein (ATX3) protein aggregations *In vivo:* ATX3Q130 *C. elegans* with ataxin-3 protein (disease) ATX3Q17 *C. elegans* with healthy protein (control)	0.1–1 mg/ml, and 2.5 mg/ml of the total *Lavado cocoa* extracts incubated for up to 96 h. 0.025–0.25 mg/ml, and 0.5 mg/ml of the polyphenolic-enriched fraction incubated for up to 96 h.	Inhibited ATX3 protein aggregations *in vitro*. STD NMR spectroscopy demonstrated a direct physical interaction between the cocoa flavanols and the polyQ tract (ATX3Q55) protein *in vitro*. Improved the mean lifespan and the motility of the disease *C. elegans* group compared to the untreated disease group. The effect was not observed in the control *C. elegans* group.	Sciandrone et al. (52)
Spinocerebellar Ataxia type 3 or Machado–Joseph disease	*In vitro:* MJD78 neuroblastoma cells (disease) MJD26 neuroblastoma cells (control) *In vivo:* Q78 transgenic drosophila (disease) Q27 transgenic drosophila (control)	3 μM of caffeic acid and resveratrol were incubated for 24, 48 h for cell-based models. 0.5 mM or 1 mM of caffeic acid, 0.25 mM of resveratrol were administered to the *in vivo* model, changing the treatments every 3 days for up to 40 days.	Both treatments reduced ROS levels, apoptosis, and mutant ATX3 in cells with increased autophagy and modulated p53 and NF-κB signaling pathways. Both treatments improved survival and locomotor activity, decreased ROS levels, and reduced mutant ATX-3. Similar effects on p53 and NF-κB were observed.	Wu et al. (53)
Spinocerebellar Ataxia type 3 or Machado–Joseph disease	Clinical trial: 14 participants	15 or 30 g of a 10% trehalose solution was administered intravenously on a weekly basis for a duration of 6 to 12 months.	The treatment was well tolerated with no serious adverse events. Neurological symptoms remained stable based on SARA score, NESSCA, 9HPT, 8MWT and WHOQOL-BREF.	Zaltzman et al. (54)
Spinocerebellar Ataxia type 3 or Machado–Joseph disease	*In vivo:* C57BL/6 mice with ATX-3 lentiviral vectors MJD transgenic mice	2% (w/v) trehalose solution in drinking water was administrated to the transgenic model for 30 weeks, and the lentiviral model for 2 or 4 weeks. Control groups received only water.	Increased autophagy and reduced the expression of mutant ATX-3 in the cells when compared to control. Improved motor function of the mouse models along with preserved cerebellar layer thickness and reduced the size of ATX-3 aggregates in Purkinje cells.	Santana et al. (55)
Spinocerebellar Ataxia type 3 or Machado–Joseph disease	*In vivo:* *SCA3* Zebrafish	50 mM of trehalose solubilised in medium was treated for 6 days post-fertilization.	Improved swimming distance of the zebrafish and increased autophagosome formation compared to vehicle treated controls. Increased in microtubule-associated protein 1A/1B light chain 3 II as an indicator of autophagy induction.	Watchon et al. (56)
Spinocerebellar Ataxia type 17	*In vitro:* 293/SH-SY5Y TBP cells SH-SY5Y TBP/Q79 cells Cerebellar slice culture derived from SCA17 transgenic mice	0.1-100 μM of trehalose, lactulose and melibiose were treated for 8 h, 48 h and 6 days in cell models. 24 mg/kg/day of trehalose, lactulose and melibiose were intraperitoneally injected to mice for 7 days.	Unlike trehalose, lactulose and melibiose could not be hydrolyzed by trehalase. All treatments reduced the polyQ protein aggregations in all cell models. All treatments enhanced autophagic activity for clearing misfolded and aggregated proteins in all cell models.	Lee et al. (57)
Spinocerebellar Ataxia type 38	Clinical trial: 10 participants	Phase 1: 600 mg/day of the algal oil derived-DHA or placebo was administered as sachets for 16 weeks. Phase 2: 600 mg/day of the algal oil derived-DHA was given to all participants for 40 weeks.	Improved in SARA scale compared to the placebo group. More significant improvement after 40 weeks of treatment, as measured by both SARA and ICARS scales. The DHA group showed improvement in cerebellar metabolism at baseline versus 40-week DHA treatment.	Manes et al. (59)
Spinocerebellar Ataxia type 38	*In vivo:* Elovl5−/− mice (disease) WT mice (control)	Mice were fed either a diet containing only PUFA precursors or a complete PUFA diet. The timing of the dietary intervention varied, from birth, at 1 month, or at 10 months of age.	Improved motor performance (measured by the balance beam test) only found when the diet was administered from birth and for 6 and 8 months. Later interventions at 1 or 10 months of age had no significant effect. The improvement of motor performance was a functional recovery, not correlate with a reduction in cerebellar atrophy.	Balbo et al. (22)

ADL, Activities of daily living; A-T, Ataxia-telangiectasia; ATX-3, Ataxin-3 protein; CMR, Cardiac magnetic resonance; CGNs, Cerebellar granule neurons; DHA, Docosahexaenoic acid; DRG, Dorsal root ganglia; DRGNs, Dorsal root ganglion neurons; ΔΨm, Mitochondrial membrane potential; EGCG, Epigallocatechin gallate; FRDA, Friedrich ataxia; FSS, Fatigue Severity Scale; FXN, Frataxin;GSH/GSSG, reduced glutathione /oxidised glutathione; H2O2, Hydrogen peroxide; NAD+, Nicotinamide adenine dinucleotide; NESSCA, the Neurological Examination Score for Spinocerebellar Ataxia; NF-κB, Nuclear Factor kappa-light-chain-enhancer of activated B cells; NMN, Nicotinamide mononucleotide; NR, Nicotinamide riboside; Nrf2, Nuclear factor erythroid 2-related factor 2; p53, Tumor protein p53; PUFA, Polyunsaturated fatty acids; PATA, Pulsed arterial tonometry assessment; PolyQ, Polyglutamine; RIPK3, Receptor-interacting protein kinase 3; ROS, Reactive Oxygen Species; SARA, Scale for the Assessment and Rating of Ataxia; SCA, Spinocerebellar Ataxia; SCAFI, the spinocerebellar ataxia functional index; SFN, sulforaphane; STD NMR, Saturation transfer difference nuclear magnetic resonance; ICARS, International Cooperative Ataxia Rating Scale; TNF-α, Tumor necrosis factor-alpha; IL-1β, Interleukin-1 beta; IL6, Interleukin-6; WHOQOL-BREF, the World Health Organization Quality-of-Life Questionnaire-BREF; WT, Wild type; 8-MWT, the 8 Meters Walking Test; 9-HPT, the 9-Hole Peg Test.

In FRDA, a study involving 34 patients treated with thiamine, also known as vitamin B1, at a dosage of 100 mg twice daily for up to 930 days demonstrated significant improvements in the Scale for the Assessment and Rating of Ataxia (SARA) score, deep tendon reflexes, swallowing, and interventricular septum thickness (33). In contrast, all forms of vitamin B3 consisting of nicotinamide adenine dinucleotide (NAD+) precursors, nicotinamide, nicotinamide mononucleotide (NMN) and nicotinamide riboside (NR), improved the survival, cardiac function and FXN protein levels via epigenetic regulation in FRDA mouse models (34, 35). However, no improvement in motor function was observed in either study (34, 35). Overall, the treatments were well-tolerated; however, one case reported severe side effects in a 40-year-old woman following the administration of 4.4 g of nicotinamide (36). Treatment involving NAD + precursors such as NR and NMN has also been tested in both *in vitro* and *in vivo* models of A-T (37, 38). Treated patient fibroblasts were found to have increased NAD + levels as well as decreased levels of mitochondrial reactive oxygen species and senescence markers (37). Similar effects were validated in the *C. elegans* model where improved locomotion and memory were noted based observational tests (i.e., swimming movement, pharyngeal pumping and chemotaxis assays) (38); Treated mice also found to have enhance survival rate, motor function based on the rotarod analysis, memory through the Y-maze spontaneous alternation test, and mitochondrial function via increased ATP levels and oxygen consumption rate (37, 38). These studies suggest that elevating NAD + levels can benefit motor function, memory and survival. Calcitriol supplementation, the active form of vitamin D, has demonstrated benefits in increasing FXN protein levels in *in vitro* and clinical studies of FRDA (30, 39). In cell models, calcitriol was found to restore mitochondrial function and enhance neuronal cell viability (39). However, it is notable that no neurological improvements were observed in clinical trials (30). Tocotrienol is a natural form of vitamin E abundant in plant oils, cocoa butter, barley and wheat germ (40). In FRDA cell models, vitamin E was found to help to prevent cell death by restoring calcium homeostasis in cardiomyocytes (10), while in human studies, it demonstrated improvements in cardiac function, lipid profiles and biomarkers related to oxidative stress and inflammation (41). Recently, butyrate supplementation, a short-chain fatty acid produced primarily by the fermentation of dietary fibre, was shown to restore butyrate-producing gut bacteria and improve adipocyte metabolism, mitochondrial respiration and glucose tolerance of the FRDA mice (42). These findings support the application of butyrate supplementation to mitigate diabetes-related symptoms in individuals with FRDA. Epigallocatechin gallate (EGCG), a polyphenol derived from green tea, has shown to restore β-tubulin and Contactin-1 expression, thereby mitigating neuronal developmental delays in FRDA mice and potentially help improve the motor and cognitive issues faced by affected individuals (43). A natural Nrf2 inducer, sulforaphane has been reported to increase FXN expression, elevate Nrf2 levels and the expression of downstream Phase II redox enzymes in cells derived from FRDA mice and FRDA fibroblasts; thus revealing its potential in targeting multiple pathological processes responsible for the disease symptoms (44–48).

There is no doubt that vitamin E has been extensively tested in AVED models. In dorsal root ganglion-derived sensory neurons of vitamin E-deficient mice, high-dose α-tocopheryl acetate supplementation successfully prevented the molecular and functional changes seen in cells and improved mechanosensation (49). In another study, natural and synthetic forms of α-tocopherol were able to increase α-tocopherol levels in the brain, heart, and lung tissues; however, high doses of synthetic α-tocopherol may negatively impact the expression of myelin genes which may impair neural synapses and consequently, motor and cognitive function (50). This underscores the importance of exercising caution with dosage Similarly, in a single case of AVED, daily consumption of 1.8 g of vitamin E successfully maintained plasma α-tocopherol levels within the normal range, with no signs of disease progression detected (51). These findings support the notion that clinical and histological phenotypes can be improved with early supplementation of vitamin E, although further research is needed to fully understand the optimal vitamin E intake.

In SCA3, phytochemicals such as *Lavado cocoa* extracts, caffeic acid and resveratrol exerted therapeutic benefits in survival and motor function through pleiotropic activities such as enhancing anti-oxidant defenses, downregulating pro-inflammatory markers and supporting mitochondrial functioning (52, 53). Trehalose, a naturally occurring sugar used as a sweetener, has been extensively studied in various models of SCA3 and SCA17 (54–57). Several have evaluated the safety and efficacy of trehalose in healthy subjects and patients with SCAs and in animal models, administered both orally and intravenously (54–57). The formation of misfolded and aggregated proteins in neurons is a hallmark of common types of SCA caused by proteins with polyQ tracts (2). Trehalose supplementation has been shown to effectively reduce the size of these protein aggregates, and improved motor function of the animal models (55–57). Additionally, dietary trehalose has garnered significant attention for its potential to stimulate the growth of health-promoting bacteria in the gastrointestinal tract (58). In SCA38, a diet rich in PUFAs (i.e., omega-3 and/or omega-6), has been shown to significantly enhance motor function in both patients and animal models, this beneficial effect does not appear to be associated with a recovery from cerebellar atrophy, as indicated by morphological measurements (22, 59). These results also highlight the importance of early intervention and diagnosis (22).

## Lifestyle interventions in the management of hereditary ataxias

The primary objective of lifestyle interventions in HA is to facilitate recovery of physiological function that enables the performance of daily routine tasks. Table 2 summarises the lifestyle related interventions such as exercise and rehabilitation programs to manage HA.

**Table 2:** Lifestyle interventions for the treatment of hereditary ataxias.

Type of hereditary ataxia	Type of study (clinical trial, in vivo, in vitro studies)	Intervention regimen	Key findings	Reference
Autosomal Recessive Hereditary Ataxias				
Friedrich Ataxia	*In vivo:* KIKO mice (disease) WT mice (control)	Voluntary wheel running or sedentary cage activity in starting at a young age (2 months) for 4 months.	Completely prevented the cardiac functional abnormalities, glucose intolerance, along with restored iron regulatory protein 1 expression compared to the sedentary KIKO mice. Improved mitochondrial function (oxygen consumption) and reduced oxidative stress in skeletal muscle without restoring FXN expression.	Zhao et al. (60)
Friedrich Ataxia	*In vivo:* FXN-depleted transgenic mice (disease) WT mice (control)	Daily treadmill running 5 days per week for 4 weeks (Gradually increase treadmill speed and incline) for both mice.	Exercise training enhanced running capacity in FXN-depleted mice but did not prevent the reduction in muscle mass or integrated stress response (ISR) activation.	Vásquez-Trincado et al. (61)
Friedrich Ataxia	Clinical trial: 39 participants	Remotely monitored their physical activity and upper extremity function using a set of wearable sensors for 7 days.	The percentage of sitting time was significantly positively correlated with FXN. Locomotion metrics demonstrated strong negative correlations with clinical score. This study supports the use of wearable sensors in assessing disease severity and monitoring motor dysfunction in FRDA.	Mishra et al. (62)
Autosomal Dominant Hereditary Ataxias				
Spinocerebellar Ataxia type 2	Clinical trial: 38 participants (19 rehabilitation group, 19 control group)	Rehabilitation group: received physical therapy occupational therapy, psychotherapy 6 h/day, 5 days/week for 24 weeks. Control group: No structured intervention; continued daily activities.	Significant improvement in total SARA score High adherence and retention rates were observed in the rehabilitation group. No significant changes were found in non-ataxia symptoms or saccadic eye movements. Patients with shorter GAG repeats responded better to therapy.	Rodríguez-Díaz et al. (63)
Spinocerebellar Ataxia type 2	Clinical trial: 1 participant	10 weeks of an integrated Ayurveda and Yoga-based lifestyle regimen.	Improvement was observed in SARA score, fall risk, and limit of stability.	Kulamarva et al. (64)
Spinocerebellar Ataxia type 3 or Machado–Joseph disease	Clinical trial: 8 participants	4 weeks of the tailored program.	Improvements in mobility, steps and ataxia severity were found after the program.	Carr et al. (65),
Spinocerebellar Ataxia type 6	Clinical trial: 12 participants (6 therapy, 6 control)	4-week home-based balance exercises with optokinetic stimuli (15 min/day, 5 days/week). The control group received no intervention.	Feasible intervention with strong test–retest reliability. Trends of improvement in balance measures but not reached statistical significance. Estimated future sample size: 80 participants per group.	Bunn et al. (66)
Spinocerebellar Ataxia type 7	Clinical trial: 1 participant	8 weeks of gait training using the ANGELLEGS exoskeleton. Each session (three 30-min sessions per week) included: Standing training (5 min) Weight-shifting exercises (5 min) Overground walking (20 min).	No adverse events were reported. Improvements were observed in SARA, BBS, and K-MBI scores after the intervention (T1), which were maintained at the four-week follow-up (T2). Improved static and dynamic stability (decrease center of pressure) at T1 and T2. Gait speed and stride length improved at T2 compared to T1. Oxygen consumption efficiency improved at T2.	Kim et al. (67)
Spinocerebellar Ataxia type 7	Clinical trial: 18 participants (7 control, 6 moderate training, 5 intensive training)	Intensive Training Group: Five weekly sessions of 2 h each for 24 weeks. Moderate Training Group: Three weekly sessions of 2 h each for 24 weeks. Control Group: No training.	Reduced SARA scores found in both moderate and intensive training groups (improved gait, stance, dysarthria). Decreased lipid damage markers (malondialdehyde, d lipohydroperoxides) and increased antioxidant enzyme maker (paraoxonase-1). No improvement in daily living activities (Barthel/Lawton scales).	Tercero-Pérez (68)
Spinocerebellar Ataxia	Clinical trial: 20 participants (10 training, 10 non-training +20 controls).	4-week cycling regimen (15 min/day, 3 days/week) A single 15-min cycling session tested for short-term effect.	Improved spinal circuitry plasticity and leg muscle coordination. Reduced ataxia severity (Reduction in ICARS scores). Normalized modulation of reciprocal inhibition.	Chang et al. (77)
Spinocerebellar Ataxia	Clinical trial: 11 participants	Modified physical therapy protocol focusing on static/dynamic balance, whole-body movements, fall prevention strategies, and falling techniques. Sessions: Twice weekly for 4 weeks (45 min each). Exercises performed without external support (progressing from minimal to no support).	Improved post-intervention balance indicated by BBS scores. Decreased fall risk based on BBS score improvements. No participants reported discomfort or suffered a fall	Santos de Oliveira et al. (88)
Spinocerebellar Ataxia	Clinical trial: 8 participants	Participants underwent a two-stage partial body weight-supported treadmill training program: 8 weeks, 50 min/session, 2 sessions/week, focusing on reducing body support and increasing treadmill speed. 10 weeks, 50 min/session, 2 sessions/week, walking without hand support and catching a ball while walking.	Improved gait performance, increased treadmill inclination, and enhanced cardiopulmonary capacity. After the second stage, improvements in balance were observed. Feasible and well-tolerated. No changes in quality of life and SARA scores.	Oliveira et al. (78)
Spinocerebellar Ataxia	Clinical trial: 33 participants (15 Verum group, 18 Sham group)	Both groups received 5-day cerebellar repetitive transcranial magnetic stimulation + physiotherapy (3 sessions/day) The sham group received placebo stimulation.	Reduced SARA scores (1.6-point improvement in verum group). Improved appendicular coordination and gait (8-Meter Walk Test) were found in the Verum group.	Grobe-Einsler et al. (79)
Hereditary Ataxias in General				
Hereditary Ataxias	Clinical trial: 20 children (Autosomal recessive HA; subtypes unspecified), 10 in each group	Intervention group: Received routine stabilization training 2 days a week and additional functional trunk training and for 1/week for 8 weeks. Control group: Received only routine stabilization training 3 days/week for 8 weeks.	Improvements in trunk control and upper limb functions (ICARS, TIST, Q-DASH) were found after training in both groups. Intervention group had improved kinetic function after training. No improvement in ICARS3 and ICARS4 scores in both groups due to lack of exercised for speech and oculomotor disorders.	Yigit et al. (69)
Hereditary Ataxias	Clinical trial: 10 participants (SCA1, SCA2, ARCA1, FRDA, Niemann-PickC)	Home-based balance and coordination training with or without vibrotactile Sensory augmentation (SA) for six weeks.	A trend in decreased SARA and SARA posture & gait scores compared to pre-training, indicating an improvement in performance after training. A significant improvement in DGI scores following the six-week training block without vibrotactile SA.	Jabri et al. (73)
Hereditary Ataxias	Clinical trial: 23 participants (SCA1, SCA3, SCA4, SCA7, SCA18, CANVAS, FRDA) on 5-week program 20 participants on 10-week program	Experimental Group: Received standard care plus a 5-week home-based CSE program (1 h/day, 5 days/week). Control Group: Received standard care only. After the initial 5-week assessment, the control group also received the 5-week CSE program.	Improved balance confidence, gait speed, quality of life and fall rate in the experimental group, at short-term (5 weeks) and long-term (10 weeks). Reduction in falls within the experimental group was found at the long-term assessment (10 weeks). No significant changes in SARA or trunk function.	Cabanas-Valdes et al. (74)
Hereditary Ataxias	Clinical trial: 76 participants (SCAs, FRDA, AOA2, ARSACS, CANVAS, ANO10, SPG7, unknown diagnosis)	Intervention Group: 6 weeks of intensive outpatient physiotherapy (land-based and aquatic) followed by a 24-week individualized home-based exercise program with fortnightly physiotherapy support. Control Group: Standard care (usual allied health and exercise).	At 7 weeks, the intervention group showed greater improvement than the control group in mFIM and SARA scores. At 30 weeks, the intervention group maintained a significant improvement in SARA scores compared to the control group, however the difference in mFIM scores was not statistically significant. Significant improvements were also found in BBS and several SF-36v2 domains at 30 weeks in the intervention group.	Milne et al. (75, 76)

AOA2, Ataxia with Oculomotor Apraxia Type 2; BBS, Berg Balance Scale; ARCA1, Autosomal recessive cerebellar ataxia type 1; ARSACS, Autosomal Recessive Spastic Ataxia of Charlevoix-Saguenay; ANO10, Autosomal Recessive Spinocerebellar Ataxia Type 10; CAG, cytosine-adenine-guanine; CANVAS, Cerebellar Ataxia, Neuropathy and Vestibular Areflexia Syndrome; CSE, Core stability exercises; DGI, Dynamic Gait Index; FRDA, Friedrich ataxia; FXN, Frataxin;, ICARS, International cooperative ataxia rating scale; ICARS3, Speech Disorder; ICARS4, Oculomotor Disorders; ISR, integrated stress response; K-MBI, Korean-Modified Barthel Index; mFIM, Functional Independence Measure; Q-DASH, Quick Disabilities of the Arm Shoulder and Hand; SA, Sensory augmentation; SARA, Scale for the Assessment and Rating of Ataxia; SCA, Spinocerebellar ataxia; SF-36v2, 36-Item Short Form Health Survey Version 2; SPG7, Spastic Paraplegia Type 7; TIST, Trunk Impairment Scale Total Score; WT, Wild Type.

In FRDA, researchers using different mouse models have observed beneficial effects of exercise in preventing cardiac functional abnormalities along with restored iron regulatory protein expression, improving mitochondrial function, reducing oxidative stress in skeletal muscle (60), and enhancing running capacity (61). While in a human study of FRDA, participants used wearable sensors to remotely monitor their physical activity and upper extremity function over a period of 7 days (62). The locomotion metrics demonstrated a strong negative correlation between total walking time/total steps and clinical scores [i.e., modified Friedreich’s Ataxia Rating Scale (mFARS), Friedreich Ataxia Rating Scale Activity of Daily Living (FA-ADL)] (62). These findings indicate that increased walking activity may improve clinical outcomes.

In SCA2, participants who received 6 h of rehabilitation per day, 5 days a week, for 24 weeks focusing on balance, coordination, and muscle strengthening showed improved SARA scores compared to those in the control group (63). However, no improvements were observed in non-ataxia symptoms or saccadic eye movements (63). Notably, the improvement in SARA scores was inversely correlated with the size of the CAG repeat expansion, suggesting that patients with shorter expansions responded better to therapy (63). Furthermore, an integrated Ayurveda and Yoga-based lifestyle regimen applied to a single patient with SCA2 has demonstrated improvements in SARA score, lowered fall risk, and stability (64). In SCA3, implementing a tailored physical activity and lifestyle program for 4 weeks improved mobility and step count while reducing ataxia severity (65). However, a four-week home-based balance training program incorporating opto-kinetic stimuli for individuals with SCA6 did not result in significant improvements in balance measures. Nevertheless, the approach was found to be feasible, with a low dropout rate (66). In a case report of an individual with SCA7, an 8-week gait training program using a robotic exoskeleton demonstrated overall improvements in both motor and cardiac function (67). Moreover, a 24-week physical rehabilitation program, including both moderate and intensive training, led to reductions in SARA scores in participants with SCA7, indicating improvements in cerebellar symptoms such as stance, gait, dysarthria, dysmetria, and tremor (68). Physical training also decreased lipid damage biomarkers (malondialdehyde and lipohydroperoxides) while increasing the activity of the antioxidant enzyme paraoxonase-1, suggesting a beneficial effect on oxidative stress. However, no significant improvements were observed in daily living activities, as measured by the Barthel and Lawton scales (68). Similarly, various studies involving a cohort of participants, each with a different kind of HA, revealed that cycling regimen, physical therapy, treadmill training program, transcranial magnetic stimulation with physiotherapy, trunk training, intensive balance training, core stability exercise, and home-based or goal-directed rehabilitation programs significantly improved motor functions compared to control groups without any interventions (69–79).

## Discussion

Due to the lack of clinically approved treatments for HA, supportive care and symptomatic treatments are the main interventions for most of these conditions; To address significant therapeutic gaps, this review aims to explore complementary therapeutic strategies, such as dietary and lifestyle interventions to potentially improve the quality of life in affected individuals.

Natural metabolites exhibit structural diversity and bioactivity, serving as substrates for various transporter systems that facilitate their delivery to targeted intracellular sites of action (80, 81). Phytochemicals such as flavonoids (i.e., *Lavado cocoa* extracts), polyphenols (i.e., EGCG, caffeic acid and resveratrol) and isothiocyanate (i.e., sulforaphane) are important naturally occurring compounds widely distributed in plants that possess multifaceted effects. A growing amount of evidence is suggestive of their potential therapeutic roles in various types of HA (43, 46, 52, 53). A variety of vitamins, have also been shown to improve certain aspects of pathology in various autosomal recessive ataxias, such as FRDA (30, 33–35, 39), A-T (37, 38) and AVED (49–51). However, a few FRDA studies have yielded inconsistent results (30, 34–36, 39). Potential factors contributing to differences may be due to the use of varied disease models, duration of drug exposure, variations in sample sizes, and the adoption of different biomarkers in pre-clinical or clinical measurements, as listed in Table 1. Furthermore, the results of various animal studies emphasise that individuals should begin treatment as early as possible to achieve optimal improvement in neurological symptoms (22, 34, 82). Meanwhile, caution should be taken when considering the consumption of nutraceuticals and an effective and toxic dose response must be examined rigorously to determine the margin of safety (36, 50).

Recent research suggests NAD + precursors and sulforaphane as promising nutraceuticals for FRDA (34, 46). Pre-clinical studies using NAD + precursors have shown improvements in cardiac function in FRDA mouse models, although effects on motor neurological function remain unclear (34, 35). While sulforaphane also shows promise in extensive pre-clinical models for its anti-oxidant effects, clinical trials are needed to validate its efficacy in FRDA (44–48). It is worth considering integrating lifestyle interventions such as regular exercise or tailored rehabilitation programs into the drug treatment regimen of FRDA for improving the motor function aspects that do not seem to be affected by the nutraceuticals. Additionally, it is not advisable to consume phytochemical supplements without the guidance of a healthcare professional, as individuals may overlook the complex interactions between medications and supplements (83, 84). For instance, dietary polyphenols have been shown to reduce the transport of thiamine and folic acid and to alter the activity of certain drugs by interacting with drug transporters or enzymes involved in metabolic reactions, which can inhibit the absorption of other nutrients (83).

In A-T, NAD + precursors seem to be very promising in mitigating the neuropathology and improving cellular mitochondrial functioning (37, 38). Given its safety profiles observed in FRDA clinical trials and animal models at higher dosages, NAD + precursors should also be trialed in A-T patients. Similarly, mounting evidence has validated the benefits of vitamin E in treating AVED, with the need for more clinical trials to determine the optimal dosages of ɑ-tocopherol acetate in individuals with AVED (49–51).

In studies of common SCAs, trehalose has demonstrated effectiveness in clearing toxic polyQ aggregates and improving motor function in various models of SCA3 and a cell model of SCA17 (55–57). Additionally, dietary trehalose has earned significant attention for its potential to stimulate the growth of health-promoting bacteria in the gastrointestinal tract, thereby promoting beneficial immune response and metabolic homeostasis (58). However, trehalose exhibits reduced efficacy in the brain as it is hydrolsed into glucose by trehalase. A study suggests that trehalose analogs, such as lactulose and melibiose, may serve as alternative strategies to enhance therapeutic effects for their resistance to trehalase hydrolysis (57). In SCA38, foods rich in PUFAs (i.e., fatty fish, olive oil) may support overall brain health and help mitigate some of the neurodegenerative processes based on the outcomes of both animal and human studies (22, 59). Consumption of fat-soluble vitamins and a low fat and carbohydrate diet have been reported to slow the progression of neurological symptoms, which highlights the importance of early diagnosis and dietary modifications (22, 85).

As efforts to source a panacea that involves a drug intervention continue, much research has also focused on rehabilitation methods associated with lifestyle changes to address symptom management (Table 2).

In FRDA, exercise training found to effectively improved the skeletal muscle and cardiac functioning by modulating mitochondrial oxygen consumption levels, glucose tolerance, as well as restoring FXN levels (60–62). However, despite the extensive benefits of exercise reported here, existing literature has limitations in small sample size and lack of sensitive biomarkers to track disease progression and assess less apparent non-motor impairments.

Current treatments for SCAs primarily aims to manage symptoms rather than to halt disease progression. The effectiveness of these treatments may vary based on the disease’s severity and progression rate, which are influenced by repeat length (86). Understanding the relationship between repeat length and disease severity is crucial for predicting outcomes and tailoring interventions for repeat expansion related diseases. A study revealed greater benefits of lifestyle interventions as part of rehabilitation programs in improving SARA scores in patients with smaller CAG repeat expansions (63). Thus, individuals with longer expansions may require more extensive management strategies to address different clinical manifestations (63). While there is evidence indicating that exercise and rehabilitation programs are generally beneficial for individuals with SCAs, there is a need to optimise the nature and scheduling of such activities to be meaningful to individuals and that can be undertaken at their own pace (64, 69–79, 87, 88). Periodic therapy sessions can facilitate customisable home exercise regimens and offer valuable feedback on progress (69, 74). Rehabilitation professionals, including specialists, play a crucial role in assisting individuals in adjusting exercise dosages based on monitored outcomes (65). Furthermore, although high adherence and retention rates were generally observed in most of the rehabilitation programs, the use of technological aids can often further enhance participants’ compliance (63, 66, 89, 90). Activity trackers can help patients to assess their current capabilities and set achievable goals. Smartwatches with safety features such as fall detection and emergency call functionalities can be helpful (91). Wearable sensors are a feasible tool for both assessing and monitoring disease progression (62). A systematic review and meta-analysis suggest that video games, exergames, and apps may be beneficial for ataxia rehabilitation and assessment, but further research is needed to fully establish their health benefits (91). Engaging in exercises like yoga or virtual reality-based activities may be able to motivate individuals to maintain adherence and these have shown improved SARA scores (64, 90). Overall, the limited number of lifestyle intervention studies in HA warrants the need for more studies to investigate the impact of different lifestyle factors (e.g., sleep, specific sport types) alone or in combination with dietary interventions, on the daily function of individuals with HA. Additionally, small sample sizes and the lack of sensitive biomarkers in existing studies limit the capacity to accurately track post-intervention disease progression and detect non-motor impairments (63, 78). Future studies may benefit from involving more participants and incorporating long-term follow-up and objective biomarkers (e.g., neuroimaging, molecular markers).

It is important to note that there are limited studies on lifestyle interventions for HA. Therefore, for the current studies reviewed in this paper, researchers often have to combine cohorts of participants with different kinds of HAs to generate a large enough sample size to increase the statistical power. As such, insufficient data is available to appropriately characterise the efficacy of the various lifestyle interventions according to individuals with different kinds of HAs and disease severity. Consequently, it is difficult to make clinically meaningful conclusions for individual patients suffering from a particular kind of HA.

## Conclusion

This review provides an overview of the dietary and lifestyle interventions in the treatment of HAs. NAD + precursors have emerged as the most researched nutraceuticals in FRDA and A-T, demonstrating benefits in cardiac and mitochondrial function, albeit with limited effects on motor and neurological functions. These limitations may be addressed by integrating lifestyle interventions, such as regular physical activity and tailored rehabilitation programs. Trehalose and polyphenols, both of which can inhibit toxic protein aggregation in common SCAs, require clinical trials to validate their efficacy. High PUFA diets and vitamin E diets appear to be promising interventions for individuals with SCA38 and AVED, although further research is needed to determine the optimal dosage. Overall, this review illuminates the great potential of nutraceutical and lifestyle interventions in the mitigation of symptoms, and benefits in promoting health status for individuals with HA.
